# Compromised paraspeckle formation as a pathogenic factor in FUSopathies

**DOI:** 10.1093/hmg/ddt622

**Published:** 2013-12-11

**Authors:** Tatyana A. Shelkovnikova, Hannah K. Robinson, Claire Troakes, Natalia Ninkina, Vladimir L. Buchman

**Affiliations:** 1 School of Biosciences, Cardiff University, Museum Avenue, CardiffCF10 3AX, UK,; 2 Institute of Physiologically Active Compounds Russian Academy of Sciences, 1 Severniy proezd, Chernogolovka 142432, Moscow Region, Russian Federation and; 3 Department of Clinical Neuroscience and MRC London Neurodegenerative Diseases Brain Bank, Institute of Psychiatry, King’s College London, De Crespigny Park, LondonSE5 8AF, UK

## Abstract

Paraspeckles are nuclear bodies formed by a set of specialized proteins assembled on the long non-coding RNA NEAT1; they have a role in nuclear retention of hyperedited transcripts and are associated with response to cellular stress. Fused in sarcoma (FUS) protein, linked to a number of neurodegenerative disorders, is an essential paraspeckle component. We have shown that its recruitment to these nuclear structures is mediated by the N-terminal region and requires prion-like activity. FUS interacts with p54nrb/NONO, a major constituent of paraspeckles, in an RNA-dependent manner and responds in the same way as other paraspeckle proteins to alterations in cellular homeostasis such as changes in transcription rates or levels of protein methylation. FUS also regulates NEAT1 levels and paraspeckle formation in cultured cells, and FUS deficiency leads to loss of paraspeckles. Pathological gain-of-function FUS mutations might be expected to affect paraspeckle function in human diseases because mislocalized amyotrophic lateral sclerosis (ALS)-linked FUS variants sequester other paraspeckle proteins into aggregates formed in cultured cells and into neuronal inclusions in a transgenic mouse model of FUSopathy. Furthermore, we detected abundant p54nrb/NONO-positive inclusions in motor neurons of patients with familial forms of ALS caused by FUS mutations, but not in other ALS cases. Our results suggest that both loss and gain of FUS function can trigger disruption of paraspeckle assembly, which may impair protective responses in neurons and thereby contribute to the pathogenesis of FUSopathies.

## Introduction

FUS is an abundant, multifunctional RNA/DNA binding protein that contributes to various aspects of cellular RNA metabolism and executes its main functions in the cell nucleus (reviewed in 1). Initially identified as a protein involved in carcinogenesis (2), FUS was recently found to be associated with certain forms of amyotrophic lateral sclerosis (ALS), frontotemporal lobar degeneration (FTLD) and several less common neurodegenerative disorders (3–6) that can be coalesced into a group of FUSopathies. The majority of ALS-linked mutations in FUS disrupts its nuclear localization signal (NLS) and results in nuclear clearance of FUS with accumulation in the cytoplasm where it forms characteristic non-amyloid inclusions (reviewed in 3). As a consequence, both loss of nuclear function(s) and gain of toxic function(s) in the cytoplasm may compromise various cellular processes in affected neurons, primarily RNA processing (7–10), axonal transport (11) and neural transmission (12). Multiple, though fragmented, experimental evidence exists that a fraction of FUS is associated with various nuclear structures. Recent studies have demonstrated a functional association of FUS with Gemini of Cajal bodies (Gems), sites of SMN protein accumulation in the nucleus, and a loss of Gems following FUS depletion or expression of a mutant with disturbed NLS (9,10). There are indications that FUS may be associated with nuclear speckles, since it interacts with serine–arginine (SR) proteins and is involved in splicing (13,14). Recently, FUS presence in another nuclear body, the paraspeckle, was demonstrated in at least three different studies (15–17). Paraspeckles are built on the long non-coding RNA (lncRNA) NEAT1, also known as MENepsilon/beta, which assembles and spatially organizes core protein constituents of the paraspeckle—p54nrb/NONO, paraspeckle protein 1 (PSP1) and PSF (18–21). Paraspeckles are believed to participate in nuclear retention of long adenosine-to-inosine hyperedited RNAs, and in storage and rapid release of certain RNAs under stress conditions (22,23). Most recently, FUS was shown to directly bind NEAT1 (16), providing a basis for physical association of the protein with paraspeckles. Interestingly, FUS shares many similarities with paraspeckle proteins, namely RNA/DNA binding capacity, involvement in chromosomal translocations leading to malignancies (24,25), interaction with C-terminal domain of RNA polymerase II (26,27) and redistribution to the perinucleolar region upon transcription inhibition (15,28). Although paraspeckles are absent in neurons under basal conditions, their formation at the early stages of ALS, triggered by increased synthesis of NEAT1, was recently demonstrated (16), suggesting participation of paraspeckles in response to neuronal stress or damage.

Here we confirmed that FUS is a core paraspeckle protein essential for the integrity of these nuclear bodies and established possible links between its role in paraspeckles and the pathogenesis of FUSopathies. We also obtained evidence that dysfunction of other paraspeckle components may be a contributory factor in these diseases.

## RESULTS

### FUS localizes to paraspeckles via its N-terminus

**Figure 1.**
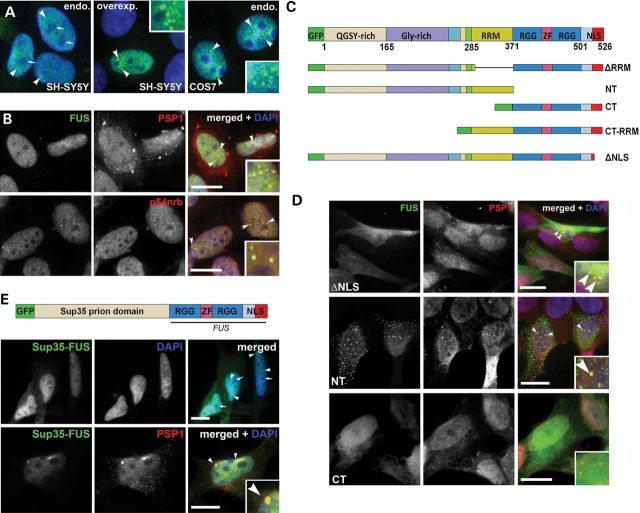
N-terminal domains of FUS are required for targeting the protein to paraspeckles. (**A**) Both endogenous and overexpressed FUS are excluded from nucleolar regions (arrows) and enriched in multiple small puncta (arrowheads) in interphase nuclei of neuroblastoma SH-SY5Y or COS7 cells. (**B**) FUS-containing nuclear dots overlap with paraspeckles (arrowheads) visualized with an antibody against PSP1 or p54nrb/NONO. (**C**) Domain organization of human FUS protein and schematic representation of constructs used in the study. (**D**) FUS lacking NLS and significantly redistributed to the cytoplasm is still enriched in paraspeckles, as is N-terminal fragment of FUS (NT), while C-terminal fragment of the protein (CT) fails to localize to paraspeckles. (**E**) Prion-like activity of FUS N-terminal domains is required for paraspeckle recruitment. Schematic map of a chimeric protein with N-terminal part of FUS replaced by the prion domain from a yeast protein Sup35 (aminoacids 1–125). Sup35–FUS localizes predominantly to the nucleus where it is excluded from nucleolar regions (arrows) and found in small puncta that overlap with paraspeckle marker PSP1 (arrowheads). Scale bars, 10 µm.

In the interphase nucleus of all cell lines examined, endogenous FUS protein forms distinct puncta and foci of various size that are clearly seen in the milieu of diffuse nucleoplasmic distribution (see Fig. 1A for SH-SY5Y and COS7 cells), suggesting highly organized subnuclear compartmentalization of the protein. green fluorescent protein (GFP)-fused full-length FUS overexpressed in these cells closely reproduces the pattern typical for the endogenous protein (Fig. 1A). Immunofluorescence with a panel of antibodies against the core proteins of known nuclear bodies was used to test the physical association of FUS with these structures in neuroblastoma SH-SY5Y cells. FUS was consistently excluded from nucleolar regions recognized by ethidium bromide staining, was not present at detectable levels in coilin-positive Cajal bodies, SMN-positive Gems or PML-positive PML bodies (Supplementary Material, Fig. S1A) but was moderately enriched in Sm antigen-positive nuclear speckles (Supplementary Material, Fig. S1A) and significantly enriched in paraspeckles detected using antibodies against PSP1 or p54nrb/NONO (Fig. 1B). Similar patterns were observed for these antibodies in COS7 and MCF7 cell lines (not shown). Even FUS with deleted NLS and significant degree of nuclear clearance was markedly enriched in paraspeckles (Fig. 1D), indicating a strong affinity of the protein to these nuclear bodies. Another ALS-associated protein, closely related to FUS, TDP-43, was also detected in paraspeckles consistent with the previous reports (15,16), although not all FUS-positive paraspeckles were positive for TDP-43 (Supplementary Material, Fig. S1B).

FUS was found in a complex with NEAT1 lncRNA (16), and it is feasible that as in the case of major paraspeckle proteins (18–21), the interaction of FUS with NEAT1 is important for its localization to paraspeckles. However, we observed that C-terminal region of FUS involved in RNA recognition and binding was not sufficient to target the protein to paraspeckles (Fig. 1C and D, panel CT). Addition of an RNA-recognition motif (RRM) to this C-terminal fragment (CT-RRM) did not change the pattern of uniformly diffuse nucleoplasmic localization of the protein and, consistently, full-length FUS lacking RRM domain only (ΔRRM) was still directed to paraspeckles (data not shown). In contrast, the N-terminal fragment of FUS was able to localize to paraspeckles on its own (Fig. 1C and D, panel NT). As this N-terminal fragment bears a potent prion-like domain (aminoacids 1–214) (29), we assessed whether prion-like activity is necessary for paraspeckle recruitment. We substituted the first 359 amino acids of FUS with a well-characterized prion domain from the yeast protein Sup35 (aminoacids 1–125) and found that the resulting chimeric protein was not only predominantly localized to the nucleus and excluded from nucleolar regions but also colocalized with paraspeckles (Fig. 1E), completely recapitulating the pattern of FUS nuclear compartmentalization. The N-terminal domain of FUS with its prion-like activity was recently proposed to cooperate with RNA-binding motifs in enabling the protein entry into non-membrane bound RNP structures such as RNA granules (30,31). Our data suggest that the paraspeckle is another RNA–protein entity requiring a protein to possess prion-like activity for its recruitment and provide further support to the role of the prion-like domain in phase transitions of FUS protein in a living cell. However, we cannot completely exclude the possibility that direct binding of FUS to NEAT1 RNA also contributes to its targeting to paraspeckles.

### FUS and other paraspeckle proteins are recruited into the same nucleolar caps

**Figure 2.**
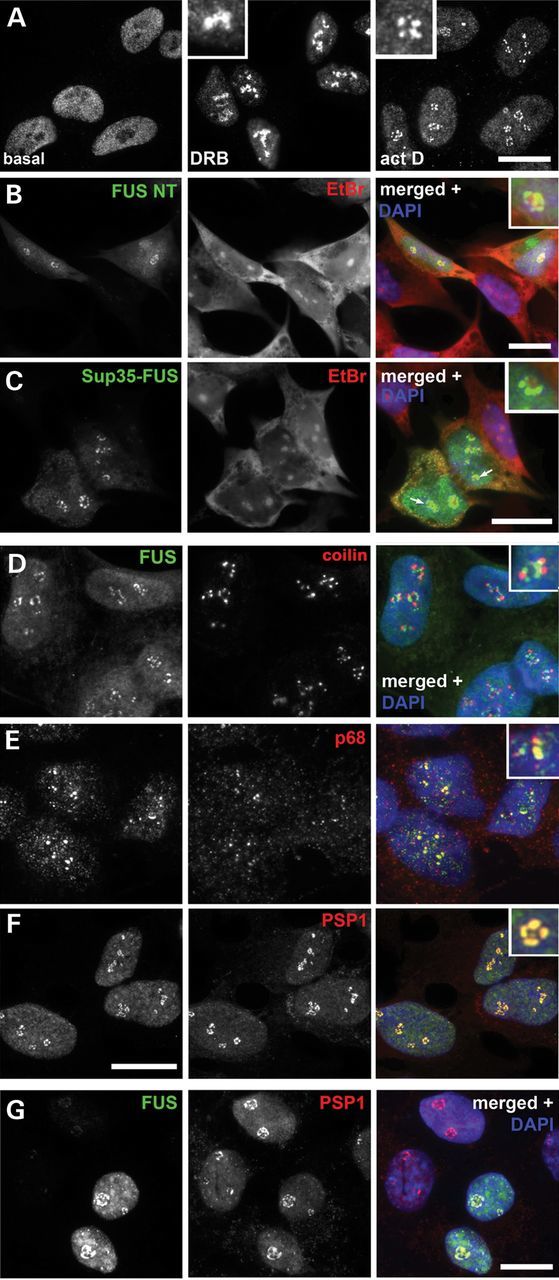
FUS is a component of nucleolar caps completely overlapping with those formed by paraspeckle proteins in neuroblastoma SH-SY5Y cells. (**A**) FUS becomes redistributed to the perinucleolar region upon treatment with DNA polymerase II inhibitor DRB but does not form nucleolar caps, in contrast to actinomycin D treatment which induces FUS recruitment to classical crescent shaped caps. (**B** and **C**) N-terminal fragment of FUS (B) and chimeric protein Sup35–FUS (C) efficiently localize to nucleolar caps upon exposure to actinomycin D. (**D**–**F**) FUS is not a component of coilin p80 caps (D), but FUS caps partially colocalize with RNA helicase p68 caps (E) and completely overlap with caps formed by PSP1 in actinomycin D-treated cells (F). (**G**) FUS is not essential for redistribution of other paraspeckle proteins to nucleolar caps, since PSP1-positive caps were observed in actinomycin D-treated cells depleted of FUS by RNA interference. Actinomycin D or DRB were added to the cells for 1.5 h prior to fixation in all experiments. Scale bars, 10 µm.

Paraspeckle proteins are known to redistribute to the perinucleolar region and become a part of dark nucleolar caps when transcription is inhibited (15,28); the same behaviour was previously reported for FUS (25). We found that classical crescent shape caps were formed by FUS only in response to global transcriptional inhibition by actinomycin D, while specific inhibition of RNA polymerase II by 5,6-dicholoro-β-d-ribofuranosylbenzimidazole (DRB) induced the protein redistribution to the perinucleolar space without formation of typical caps (Fig. 2A). The N-terminal domains of FUS are largely responsible for nucleolar cap recruitment of the protein (Fig. 2B and Supplementary Material, Fig. S2A) and this is mediated by prion-like activity since Sup35–FUS chimeric protein also readily redistributes to nucleolar caps (Fig. 2C). Thus, the prion-like domain defines FUS localization to paraspeckles and nucleolar caps. It is known however, that nucleolar caps formed by different proteins may only partially overlap (28). To determine whether FUS is a component of the same caps as other paraspeckle proteins we immunostained actinomycin D-treated cells with antibodies against paraspeckle proteins PSP1 and p54nrb/NONO, and against non-paraspeckle proteins p80 coilin and RNA helicase p68, which are also found in nucleolar caps. FUS-positive nucleolar caps were distinct from those formed by p80 coilin (Fig. 2E) and only partially overlapped with p68 (Fig. 2F) to form complex three-dimensional cap-like structures that were especially evident in COS7 cells (Supplementary Material, Fig. S2B). In contrast, complete colocalization was observed with caps formed by paraspeckle proteins PSP1 and p54nrb/NONO (Fig. 2G and Supplementary Material, Fig. S2C). A related protein, TDP-43, either endogenous or overexpressed, was not observed in nucleolar caps induced by either actinomycin D (Supplementary Material, Fig. S2D) or DRB (not shown). However, FUS does not play an essential role in the recruitment of other paraspeckle proteins to nucleolar caps since this process was not disturbed in FUS-depleted cells (Fig. 2G).

### FUS interacts with a core paraspeckle protein p54nrb/NONO in an RNA-dependent manner

**Figure 3.**
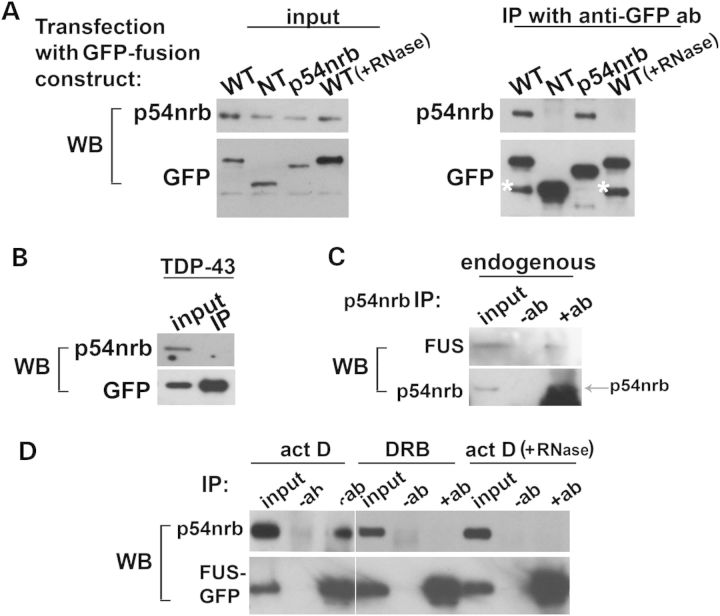
FUS associates with p54nrb/NONO *in vivo* via RNA and this interaction is regulated by ongoing transcription. (**A** and **B**) Immunoprecipitation revealed an RNase sensitive interaction of p54nrb/NONO with full-length FUS protein (FUS WT) but not with N-terminal part of FUS (FUS NT) (A) or full-length TDP-43 (B). Full-length FUS, N-terminal FUS fragment (aminoacids 1–359, NT), p54nrb/NONO or TDP-43 expressing plasmids were transfected into SH-SY5Y cells and 24 h after transfection immunoprecipitated on anti-GFP antibody coated beads. To test the role of RNA in FUS-p54nrb/NONO interaction the lysate of FUS-GFP WT transfected cells was treated with RNase A for 30 min at RT prior to incubation with beads. Asterisks mark non-specific bands. (**C**) Interaction of endogenous FUS and p54nrb/NONO proteins in COS7 cells. Co-immunoprecipitations of FUS with anti-p54nrb/NONO antibody from cell lysates. A part of very intense 50 kDa immunoglobulin heavy chain band is seen just under the p54nrb/NONO band because the same antibodies were used for immunoprecipitation and western blotting. (**D**) FUS remains associated with p54nrb/NONO in actinomycin D but not DRB treated cells. Protein complexes of full-length GFP-tagged FUS were immunoprecipitated from lysates of SH-SY5Y cells untreated or treated with inhibitors of transcription for 1.5 h.

To assess whether FUS and other paraspeckle proteins can be present in the same macromolecular complexes we carried out co-immunoprecipitation experiments with GFP-tagged FUS protein expressed in SH-SY5Y cells. GFP–FUS efficiently pulled down endogenous p54nrb/NONO but this co-immunoprecipitation was completely abolished when lysates were pretreated with RNase A, suggesting that interaction between the two proteins is RNA-dependent (Fig. 3A). Consistent with this result, the N-terminal part of FUS lacking major RNA-binding domains (NT) did not precipitate p54nrb/NONO. Despite structural and functional similarities between FUS and TDP-43, the latter was not found in complex with p54nrb/NONO (Fig. 3B). We have also demonstrated co-immunoprecipitation of endogenous FUS with endogenous p54nrb/NONO (Fig. 3C). It should be noted that these experimental approaches reveal not only paraspeckle-associated, but all *in vivo* complexes containing FUS and p54nrb/NONO. Although p54nrb/NONO is a major paraspeckle protein it has other intracellular functions, particularly in transcription (26) and FUS also has been implicated in this process (27,32), suggesting that certain transcription complexes might include both proteins. To assess whether interaction of FUS and p54nrb/NONO was preserved upon inhibition of transcription, we carried out co-immunoprecipitation of these proteins from cells treated with two mechanistically different inhibitors. A significant fraction of GFP-labelled FUS was still associated with p54nrb/NONO in cells treated with actinomycin D but not DRB and similarly to untreated cells, this interaction was RNA-dependent (Fig. 3D). As both FUS and p54nrb/NONO are able to interact with the C-terminal domain of RNA polymerase II (26,27), a plausible explanation of these data is that while the FUS–p54nrb/NONO interaction remains intact when the intercalating agent actinomycin D stalls the RNA polymerase complex and prevents elongation, this interaction becomes impaired when the assembly of a transcription unit containing FUS and p54nrb/NONO is blocked by DRB, an inhibitor of Cdk9 and other kinases that regulate integrity and activity of transcriptional complexes (33). Taken together, our data suggest that these proteins are components of the same transcriptional complex(es) and come into contact co-transcriptionally via interaction with RNAs present in these complexes.

### Methylation regulates paraspeckle protein distribution in the nucleus

**Figure 4.**
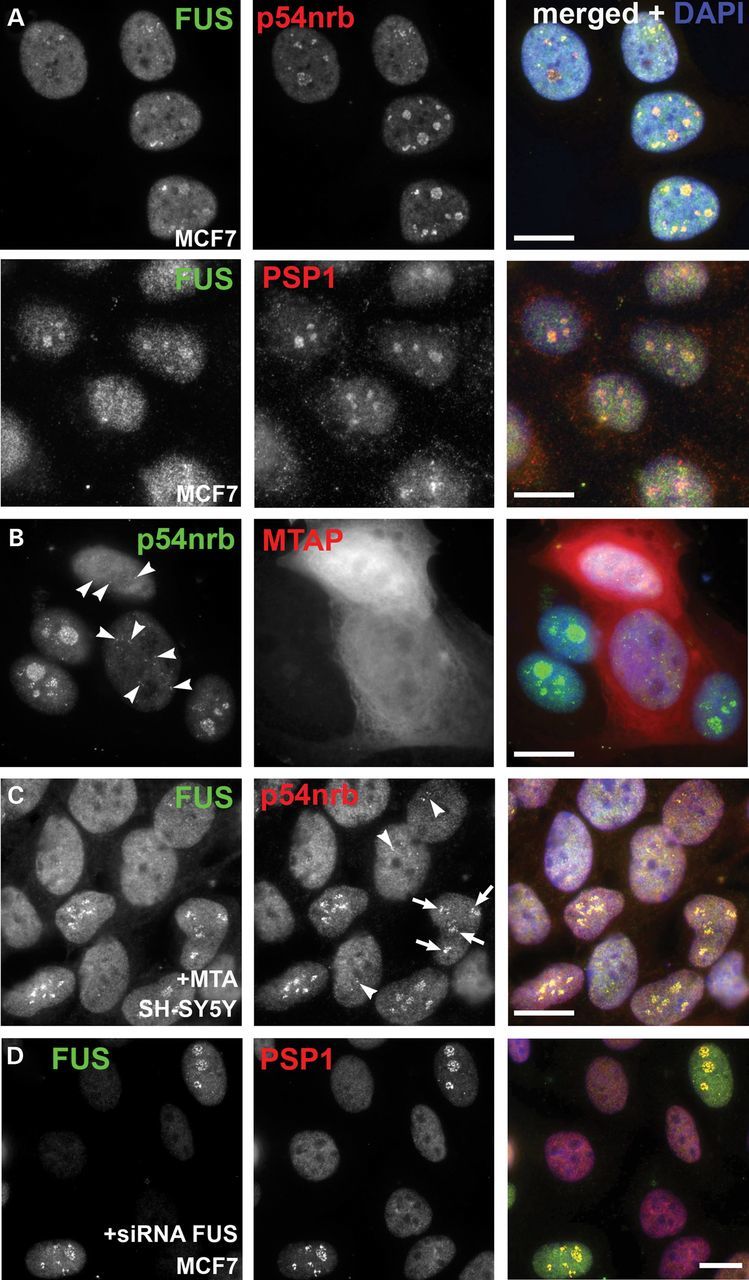
Protein methylation regulates distribution of paraspeckle proteins in the nucleus. (**A**) Paraspeckle proteins p54nrb/NONO, PSP1 and FUS accumulate in the perinucleolar region in the majority of MCF7 breast cancer cells under basal conditions. (**B**) Ectopic expression of Flag-tagged MTAP protein in MCF7 cells restores paraspeckle distribution of endogenous p54nrb/NONO protein. Cells were fixed and processed for staining 24 h post-transfection. Arrows show p54nrb/NONO-positive paraspeckles in MTAP-expressing cells. (**C**) Prolonged (24 h) treatment of neuroblastoma SH-SY5Y cells with MTA, a global inhibitor of protein methyltransferases, induces redistribution of p54nrb/NONO and FUS into perinucleolar region in a fraction of cells (arrows). Paraspeckles are still preserved in cells where such redistribution did not occur (arrowheads). (**D**) Perinucleolar localization of PSP1 in MCF7 cells is abolished by siRNA knockdown of FUS expression. Scale bars, 10 µm.

In our experiments with MCF7 breast cancer cell line we found FUS and other paraspeckle proteins localized predominantly in the perinucleolar region in the vast majority of cells (Fig. 4A), a pattern strikingly different to distribution of these proteins in the nucleus of other types of cultured cells. It has been previously demonstrated that this cell line lacks methylthioadenosine phosphorylase (MTAP) gene which affects the methionine salvage pathway and therefore leads to decreased levels of protein methylation (34). Moreover, coilin p80 is localized to the perinucleolar region in a fraction of MCF7 cells and this fraction is increased following inhibition of protein methyltransferases (35). We hypothesized that this state of hypomethylation is responsible for triggering the relocalization of paraspeckle proteins to perinucleolar regions in these cells. To test this we first performed a rescue experiment in MCF7 cells by ectopically expressing Flag-tagged MTAP. Indeed, in MTAP-expressing cells paraspeckles, visualized by anti-p54nrb/NONO staining, were restored and perinucleolar localization of paraspeckle proteins was no longer detected (Fig. 4B). Furthermore, when neuroblastoma SH-SY5Y cells characterized by normal paraspeckle protein distribution were treated for 24 h with methylthioadenosine (MTA), a global methyltransferase inhibitor, a fraction of cells displayed relocation of paraspeckle proteins to the perinucleolar region (Fig. 4C, arrows). The same result was obtained in cells overexpressing GFP-fusion full-length FUS protein (Supplementary Material, Fig. S2E). Following MTA treatment, paraspeckles were preserved in neuroblastoma cells with conventional FUS distribution (Fig. 4C, arrowheads), but not in those with the protein redistributed to the perinucleolar region. Arginines in FUS are frequently dimethylated (36,37) while this has not been observed for core paraspeckle proteins p54nrb/NONO, PSF and PSP1. Therefore it is plausible that the methylation state of FUS and other essential paraspeckle proteins known to be methylated, such as hnRNP K (38), regulates relocation of non-methylatable paraspeckle proteins to the perinucleolar region. In support of this idea, nucleolar targeting of paraspeckle proteins was no longer detected in MCF7 cells with significantly decreased FUS levels achieved by siRNA knockdown (Fig. 4D). Changes in FUS methylation have been linked to the development of FUSopathies via impaired nuclear transport of methylated ALS-associated variants and their entrapment in the cytoplasm (39). In FTLD-FUS, the gene encoding FUS protein is not mutated but hypomethylation of the protein leads to its redistribution to the cytoplasm (39) where it forms pathological inclusions (5). Since a hypomethylated state dramatically affects normal distribution of FUS and other paraspeckle proteins in the nucleus, an abnormal decrease in methylation levels at early stages of FTLD-FUS might disrupt the association of FUS and other paraspeckle proteins with NEAT1 and each other, impairing paraspeckle assembly and consequently, cellular protective responses.

### Cellular level of FUS regulates paraspeckle assembly and maintenance

**Figure 5.**
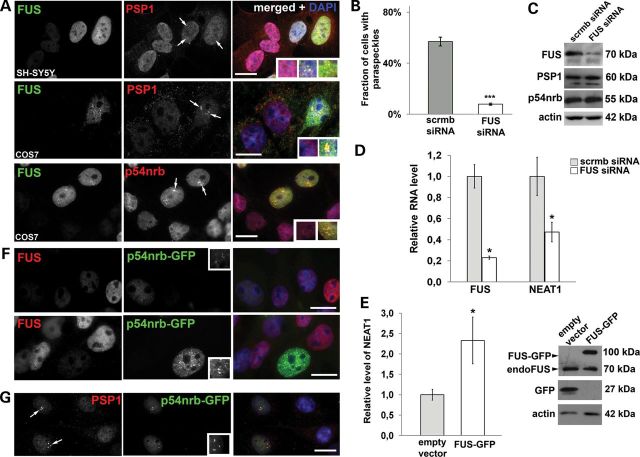
FUS is important for the integrity of paraspeckles and regulates NEAT1 levels. (**A**–**C**) siRNA knockdown of FUS causes loss of paraspeckles in cultured cells as visualized by immunostaining for core paraspeckle proteins PSP1 and p54nrb/NONO (**A** and **B**) without alterations in total levels of these proteins (**C**). Representative images for PSP1 and p54nrb/NONO distribution in COS7 and SH-SY5Y cells and quantification for COS7 cells are shown. Anti-PSP1 staining was used to visualize paraspeckles for counts. Arrows indicate paraspeckles preserved in cells with normal FUS levels. (**D** and **E**) FUS protein levels regulate abundance of long non-coding RNA NEAT1. Downregulation of FUS expression by siRNA knockdown significantly decreases NEAT1 levels (D), while FUS overexpression results in elevated NEAT1 (E) in MCF7 cells as measured by qPCR with primers specific for both short (NEAT1_1) and long (NEAT1_2) isoforms of NEAT1. Cells were transfected with either empty pEGFP-C1 vector or GFP-FUS and analysed 24 h post-transfection. Western blotting with anti-FUS antibody shows approximately equal levels of FUS-GFP and endogenous FUS in total cell culture lysates, considering that efficiency of transfection of MCF7 cells was ∼25%, transfected cells expressed approximately 4 times more ectopic than endogenous FUS protein. (**F** and **G**) p54nrb/NONO substitutes for loss of FUS function required for paraspeckle formation. GFP fused p54nrb/NONO expressed in FUS-depleted COS7 cells formed multiple paraspeckle-like structures in dose-dependent manner (F), and these were positive for PSP1 (G, arrows**).** In all experiments cells were transfected with either a pool of siRNA specifically targeting FUS protein (FUS siRNA) or scrambled siRNA (scrmb siRNA) and analysed 72 h post-transfection. *P < 0.05 and ***P < 0.001 (Mann–Whitney *U*-test). Error bars represent SEM. Scale bars, 10 µm.

ALS-associated mutations can potentially exert a deleterious effect via two mechanisms—loss of nuclear function and gain of toxic function in the cytoplasm. To determine the consequences of FUS deficiency we knocked down FUS expression by RNA interference. Using a pool of FUS targeting siRNAs we achieved ∼75% knockdown at the level of mRNA and 50–70% at the level of protein in COS7, MCF7 or SH-SY5Y cells at 72 h post-transfection (see Fig. 5B and C for MCF7 cell line). Despite the levels of paraspeckle proteins PSP1 and p54nrb/NONO remaining unchanged upon FUS knockdown (Fig. 5C), paraspeckles disappeared from FUS-depleted cells (Fig. 5A). To assess whether this effect was because of decreased levels of NEAT1 lncRNA we performed qRT-PCR with primers that simultaneously detect both long (NEAT1_2) and short (NEAT1_1) isoforms of NEAT1. NEAT1 levels were significantly lower in MCF7 cells treated with FUS siRNA compared with scrambled siRNA control (Fig. 5D). NEAT1 downregulation was previously observed upon knockdown of p54nrb/NONO and PSF (20), and therefore, it is likely that FUS contributes to maintenance of the steady-state level of NEAT1 transcripts in the same way as these major paraspeckle proteins. The effect of FUS knockdown on paraspeckles is seemed to be rescued by overexpression of another paraspeckle protein as multiple paraspeckle-like and PSP1-positive structures reappear in COS7 cells with dramatically reduced FUS levels that are expressing GFP-p54nrb/NONO (Fig. 5F and G). We cannot completely exclude that these structures represent small p54nrb/NONO aggregates that also contain PSP1 because of high affinity heterodimerisation of these two proteins (19,40). However, these structures were observed in FUS-depleted cells with a low level of GFP-p54nrb/NONO expression that does not induce aggregate formation in naïve cells, making our suggestion that the protein supports formation of physiological structures, i.e. paraspecles, very credible. The observed effect could not be attributed to restoration of NEAT1 levels, since GFP-p54nrb/NONO expression neither altered NEAT1 levels in cells with normal FUS expression nor did it rescue the decrease in NEAT1 levels in the cells upon FUS knockdown (Supplementary Material, Fig. S3). Therefore, p54nrb/NONO is a good candidate for substituting the architectural role of FUS in paraspeckles. Furthermore, we observed that in contrast to p54nrb/NONO, expression of FUS–GFP does lead to a statistically significant elevation of NEAT1 levels (Fig. 5E).

### Mislocalized FUS traps paraspeckle proteins in cytoplasmic aggregates in cultured cells

**Figure 6.**
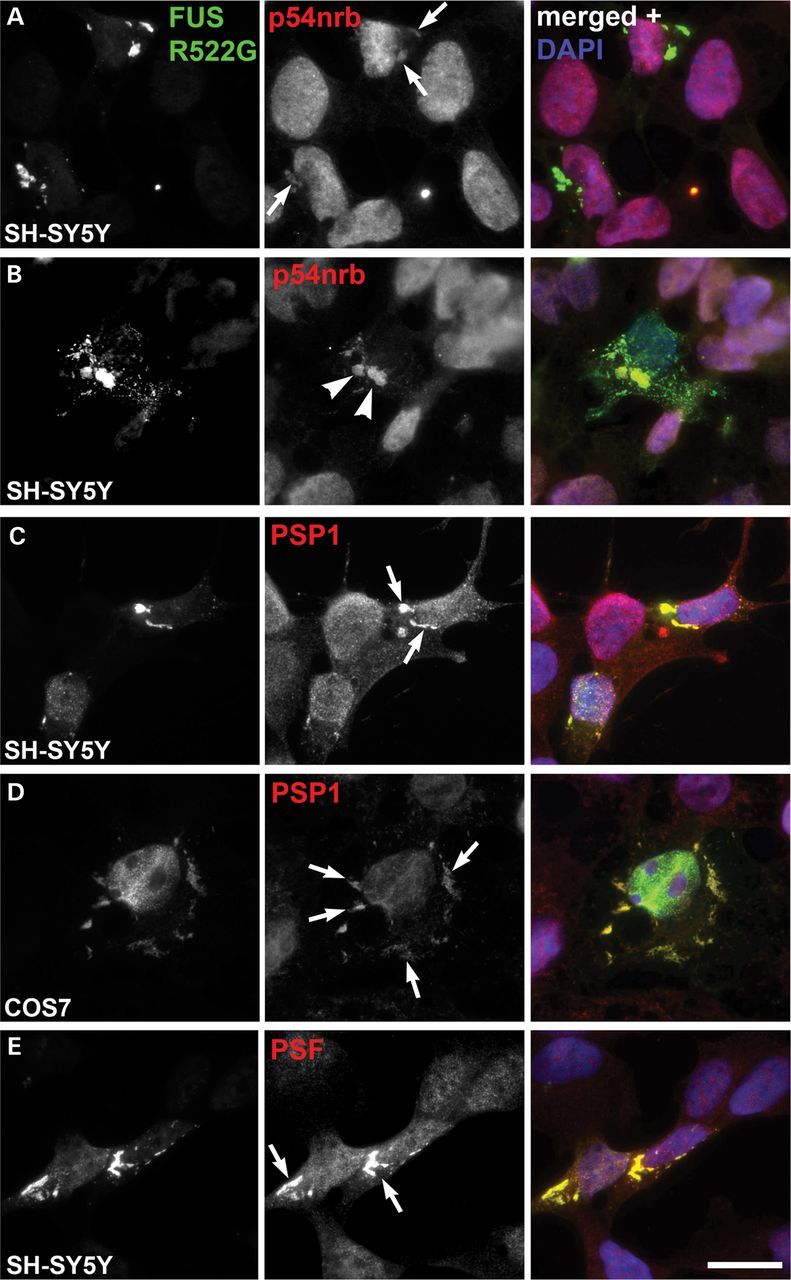
Cytosolic FUS aggregates sequester paraspeckle proteins. p54nrb/NONO protein is consistently present in aggregates formed by cytoplasmically mislocalized FUS bearing an ALS-linked R522G substitution (**A**, arrows), and in some cells p54nrb/NONO is cleared from the nucleus and accumulates in FUS aggregates (**B**, arrowheads)**.** Core paraspeckle proteins PSP1 and PSF are recruited into FUS aggregates in neuroblastoma SH-SY5Y (**C** and **E**, arrows) and COS7 (**D**, arrows) cells. Cells were analysed 24 h post-transfection. Scale bar, 10 µm.

To model toxic gain of function of ALS-associated FUS mutations, we expressed FUS variants with impaired NLS in cultured cells. Despite mislocalization to the cytoplasm, in cells expressing low levels of a mutant protein all FUS variants tested including truncated FUS lacking 60 C-terminal amino acids (p.G466VfsX14), were still significantly enriched in paraspeckles further confirming the high affinity of FUS for these nuclear bodies (Supplementary Material, Fig. S4A and B). However, in cells accumulating high levels of mutant FUS protein multiple cytoplasmic aggregates are formed and in SH-SY5Y cells expressing FUS R522G p54nrb/NONO was consistently (in 80.3 ± 0.96% of all cells with aggregates) present in these aggregates (Fig. 6A, arrows). Similar results were obtained for ΔNLS or p.G466VfsX14 constructs (data not shown). This result was unexpected as p54nrb/NONO is usually almost entirely restricted to the nucleus in cultured cells. Furthermore, in a small fraction of cells, a significant amount of p54nrb/NONO was detected in the aggregates together with a marked clearance of the protein from the cell nucleus (Fig. 6B). PSP1 and another core paraspeckle protein, PSF (polypyrimidine tract-binding protein-associated splicing factor), also accumulated within the cytosolic FUS aggregates in SH-SY5Y and COS7 cells (Fig. 6C–E, arrows). In contrast, we failed to detect p54nrb/NONO in cytoplasmic aggregates formed by 25 kDa C-terminal fragment of TDP-43, and multiple dot-like nuclear aggregates of this protein did not overlap with paraspeckles visualized with p54nrb/NONO (Supplementary Material, Fig. S4C, arrowheads).

### P54nrb/NONO accumulates in nuclear and rarely cytoplasmic inclusions formed by truncated FUS in a transgenic mouse model

**Figure 7.**
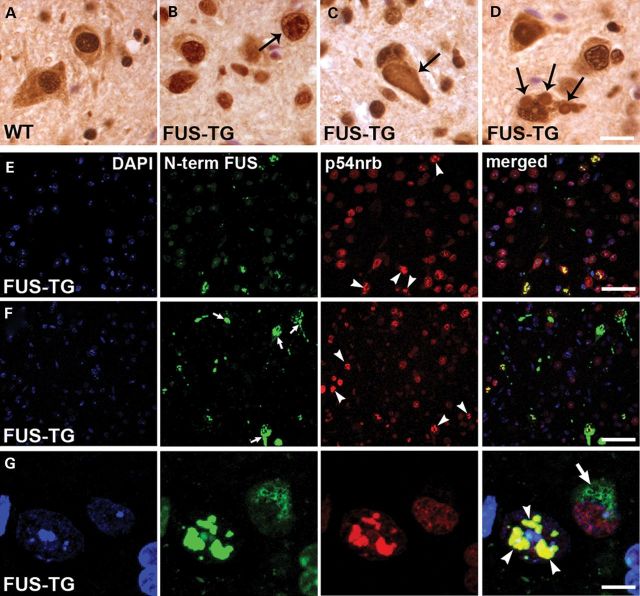
p54nrb/NONO is sequestered into cytoplasmic and nuclear inclusions formed by truncated FUS in a transgenic mouse model of FUSopathy. (**A**) Immunohistochemical staining with antibody against p54nrb/NONO shows that the protein accumulates in the cytoplasm of large spinal motor neurons but in the cytoplasm of small neurons and glial cells of non-transgenic mice. (**B**–**G**) p54nrb/NONO is detected in virtually all nuclear FUS aggregates by double immunofluorescence (arrowheads in higher magnification image shown in G). Occasionally p54nrb/NONO is also detected by immunohistochemistry in cytoplasmic inclusions (C and D, arrows) formed by truncated FUS protein in spinal motor neurons of symptomatic FUS-TG mice. Both truncated and endogenous FUS were visualized by an antibody recognizing an N-terminal FUS epitope (N-term FUS) present in both proteins. Scale bars, A–D: 15 µm; E and F: 35 µm; G: 10 µm.

Recently we produced and characterized a transgenic mouse model of FUSopathy (FUS-TG mouse line) based on aggregation of C-terminally truncated human FUS (41). In these mice human FUS protein identical to the NT variant shown in Figure 1C, but without GFP tag, forms multiple cytoplasmic and nuclear inclusions in selected neuronal populations. Moreover, the recruitment of endogenous mouse FUS to these inclusions, particularly those within the nucleus, is observed. We hypothesized that paraspeckle proteins might be recruited into FUS inclusions in this transgenic mouse model through an interaction with endogenous FUS. First we showed that in mouse nervous tissues p54nrb/NONO is predominantly nuclear, although in some cells, particularly in large motor neurons, a large fraction of the protein is also found in the cytoplasm (Fig. 7A). Co-staining spinal cord sections from symptomatic FUS-TG mice with anti-p54nrb/NONO and N-terminal specific FUS antibodies revealed a strong p54nrb/NONO immunoreactivity and complete overlap with anti-FUS staining for virtually all nuclear FUS aggregates in spinal motor neurons (Fig. 7E–G). These nuclear inclusions were also evident in conventional immunohistochemistry using detection of the signal with 3,3′-diaminobenzidine as a substrate (Fig. 7B). With the higher sensitivity provided by this method compared with immunofluorescence, rare p54nrb/NONO-positive cytoplasmic inclusions were also detected (Fig. 7C and D). PSF was also detected in a fraction of nuclear but not cytoplasmic FUS inclusions, which is consistent with its predominantly nuclear localization in neurons (Supplementary Material, Fig. S5B and C). In contrast, PSP1 was found neither in nuclear nor in cytoplasmic FUS aggregates, although like p54nrb/NONO it is also abundant in neuronal cytoplasm (Supplementary Material, Fig. S5A). Since aggregation of truncated FUS occurs extremely rapidly (41), probably after reaching a certain concentration threshold, p54nrb/NONO and PSF are more efficiently recruited into aggregates in the nucleus where they predominantly reside, while their lower levels in the cytoplasm allow formation of cytoplasmic inclusions only in a fraction of neurons. Potentially, paraspeckles formed in motor neurons of FUS-TG mice in response to damaging effects of accumulating exogenous protein might become seeding centres for aggregation of truncated FUS, which sequester endogenous FUS-p54nrb/NONO-PSF complexes. These aggregation ‘cores’ may subsequently grow and fuse to each other to give rise to nuclear inclusions. The fact that unlike two other core paraspeckle proteins, PSP1 is not detected in final products of FUS aggregation in neurons of transgenic mice indicates a certain selectivity of paraspeckle protein co-aggregation and not mere entrapment of entire paraspeckles.

### P54nrb/NONO-positive inclusions are abundant in spinal motor neurons of ALS-FUS patients but not healthy controls or other ALS cases

**Figure 8.**
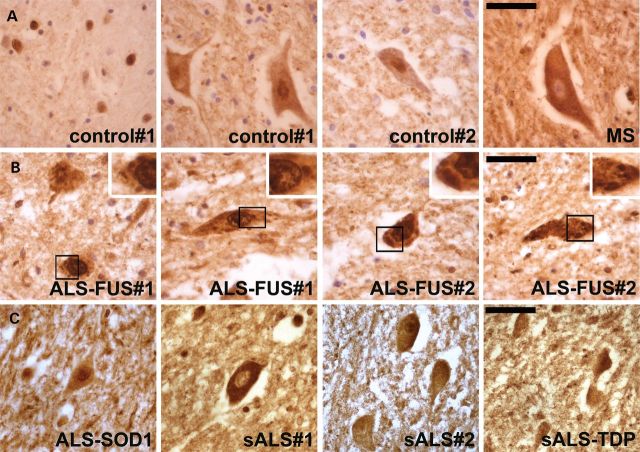
p54nrb/NONO is a constituent of cytoplasmic and nuclear inclusions in human familial ALS-FUS. (**A**) p54nrb/NONO is confined to the nucleus in the majority of glial cells and small neurons in the spinal cord of non-ALS individuals (control#1). However, in spinal motor neurons p54nrb/NONO is present at considerable levels in the cytoplasm. Representative images of spinal motor neurons from two healthy individuals and one MS case stained with anti-p54nrb/NONO antibody are shown. (**B**) Multiple nuclear and cytoplasmic p54nrb/NONO-positive inclusions are detected in two familial ALS cases with FUS mutations (ALS-FUS). (**C**) p54nrb/NONO is diffusely distributed in the nucleus and cytoplasm of sporadic ALS (sALS) cases, including a case with confirmed TDP-43 inclusions (sALS-TDP), as well as in a case of familial ALS with SOD1 mutation (ALS-SOD1). Scale bars, 30 µm.

The observed redistribution of p54nrb/NONO protein in cell culture and in a transgenic mouse model suggested that this protein could be sequestered into pathological aggregates in human FUSopathies. We therefore used immunohistochemistry to evaluate the distribution of p54nrb/NONO in the spinal cord of ALS-FUS patients compared with neurologically healthy controls, a case of multiple sclerosis (MS) and sporadic ALS (sALS) cases. p54nrb/NONO was nuclear in the majority of small neurons and glial cells but strikingly, displayed prominent cytoplasmic staining in many motor neurons from non-ALS individuals (Fig. 8A), and in some of these neurons was completely excluded from the nucleus (Fig. 8A, MS case). Multiple p54nrb/NONO immunoreactive nuclear and cytoplasmic inclusions of various sizes were noted in surviving motor neurons but not glial cells in two studied ALS-FUS cases (Fig. 8B). Such structures were not detected in any of three control non-ALS subjects (Fig. 8A), nor in three sALS cases including one with confirmed presence of TDP-43 inclusions (Fig. 8C, sALS-TDP), nor in an ALS-SOD1 case (Fig. 8C, ALS-SOD1).

## DISCUSSION

Paraspeckles are nuclear structures with poorly understood functions, although their importance for selective nuclear retention of hyperedited transcripts, selective storage of certain RNAs and their rapid release under stress conditions have been demonstrated (22,23). Paraspeckles are present in the nucleus of almost all cultured cells (42), suggesting that *in vitro* conditions favour their assembly and function. However, they are absent in embryonic stem cells (43) and their presence in mammalian tissues is subpopulation-specific. Generally, these structures are abundant in tissues with a high level of expression of the long non-coding RNA NEAT1, for example, in cells of surface gastric epithelium (44). Ablation of NEAT1 synthesis in mice leading to the loss of paraspeckles does not result in any detectable abnormalities in animal development (44), which is surprising because, perceivably, preventing synthesis of defective proteins from hyperedited transcripts is important for cellular homeostasis. It is feasible that NEAT1 synthesis and paraspeckle assembly are constitutive in some types of cells but in other types of cells occur transiently in response to specific stimuli, such as certain types of stress (45), and that developmental mechanisms able to compensate for the loss of paraspeckle function become activated in the knockdown model. Although under normal conditions the level of NEAT1 is low in the nervous tissue and prominent paraspeckles are absent from neurons (44), enhanced NEAT1 expression and nucleation of paraspeckles have been recently observed in motor neurons at early stages of ALS development (16). One interpretation of this finding is that formation of paraspeckles represents an early protective response of these neurons to some deleterious insults. If so, events/factors impairing this pathway would weaken or even remove this protective barrier. Several lines of evidence led us to suggest that such impairment may contribute to the development of neuronal dysfunction in human FUSopathies.

We demonstrated that FUS is not only an integral component of paraspeckles, as has been shown previously (15), but also significantly contributes to their stability by both regulating NEAT1 steady-state levels and maintaining the structure of this nuclear body. Therefore, nuclear deficiency of FUS typical of FUSopathies may impede paraspeckle formation needed for an adequate response to stress. Furthermore, our data obtained in cultured cells, transgenic mice and human post-mortem tissue indicate that in addition to the loss of its own nuclear function, FUS aggregation might cause sequestration of paraspeckle components into pathological inclusions. One of them, p54nrb/NONO, has been implicated in multiple and diverse cellular functions, including splicing regulation (46,47), DNA unwinding (48), targeting DNA binding proteins to their binding sites (14), transcription termination (49), DNA repair (50) and circadian rhythm maintenance (51). Therefore, its entrapment in inclusions and subsequent withdrawal from cellular metabolism would be expected to negatively affect these pathways and may contribute the progression of pathology independently of a direct effect on paraspeckle formation.

**Figure 9.**
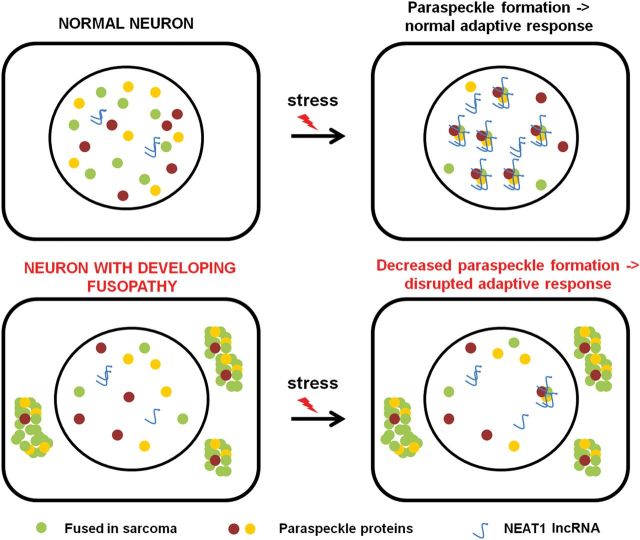
A hypothetical scheme describing how mislocalization of FUS protein typical for human FUSopathies may disrupt early response of neurons to stressful conditions because of compromised paraspeckle formation.

Taken together results of our studies support a model of disrupted protective function of paraspeckles triggered by FUS mislocalization and aggregation (Fig. 9). According to this model, neuronal cells respond to deleterious external and internal factors by activation of a protective mechanism leading to upregulation of NEAT1 levels and formation of paraspeckles. However, in conditions of nuclear FUS deficiency occurring in FUSopathies, the high level of NEAT1 required for their assembly cannot be achieved or maintained. Moreover, FUS abnormally deposited in the cytoplasm sequesters other paraspeckle proteins decreasing their nuclear pool. As a result of the deficiency of key structural elements at the early stages of FUSopathy development, the assembly of paraspeckles is compromised and the paraspeckle-based response is impaired, which contributes to progression of neuronal pathology.

It is becoming increasingly recognized that paraspeckle function becomes important under conditions of stress (44,45), and impairment of the paraspeckle response may lead to dramatic consequences, especially in long-living cells such as neurons. We have demonstrated that deregulation of FUS protein, a structural component of paraspeckles, caused by mutation or post-translational modifications associated with certain types of neurodegeneration, can perturb this pathway. So far, FUS is the only paraspeckle protein directly linked to neurological pathology; in the present study we demonstrated the involvement of other components of these nuclear bodies in FUS-mediated neurodegeneration. Interestingly, the presence of PSF in the insoluble proteome in FTLD brains has been recently reported (52). These observations suggest the need for further studies of the role of paraspeckle components in the pathogenesis of FUSopathies, and scrutiny of their ability to cause neuronal pathology independently of FUS.

## MATERIALS AND METHODS

### Expression plasmids, transfection and treatments

DNA fragments encoding full-length FUS, TDP-43, p54nrb/NONO and MTAP, truncated FUS variants, FUS variants carrying mutations, and prion domain of Sup35 protein were produced by RT-PCR amplification with SuperScript III and AccuPrime polymerases (Invitrogen) from human (SH-SY5Y cells) total RNA using corresponding primers, cloned into pCR-BluntII-TOPO vector (Invitrogen). After sequence validation DNA fragments subcloned into the pEGFP-C1 vector (Clontech) downstream of and in-frame with GFP or in pFlag-CMV4 vector (Sigma). SH-SY5Y human neuroblastoma cells, COS7 and MCF7 cells were maintained in Dulbecco's modified Eagle's medium (Invitrogen), supplemented with 10% fetal bovine serum. For immunofluorescence cells were grown on poly-l-lysine coated coverslips. Cells were transfected with expression plasmids and/or FUS siRNA using Lipofectamine2000 (Invitrogen) according to the manufacturer’'s instructions. siRNA-mediated knockdown of endogenous FUS was achieved using FUS-specific SiGENOME SMART pool M-009497-02 (target sequences: 5′-ccuacggacagcagaguua-3′, 5′-gauuauacccaacaagcaa-3′, 5′-gaucaauccuccaugagua-3′, 5′-cgggacagcccaugauuaa-3′) (Thermo Scientific). As a control for off-target effects, non-specific scrambled siRNA (target sequence: 5′-ggacuaauaguugugcuccaauuua-3′) (Invitrogen) was used. To block transcription cells were treated with 5 µg/ml actinomycin D (Calbiochem) or 25 μg/ml DRB(Sigma) for 2 h. To decrease levels of protein methylation 5′-deoxy-5′-methylthioadenosine (MTA) was applied to SH-SY5Y cells in full medium at a final concentration of 750 µM for 24 h. For nucleolus staining living cells were exposed to 10 µg/ml of ethidium bromide for 2 hs prior to fixation.

### Immunofluorescence on coverslips

Cells were fixed with 4% paraformaldehyde on ice for 15 min, followed by washes with PBS and 5 min permeabilization in cold methanol. After three washes with PBS and blocking in 5% goat serum/PBS/0.1% Triton X-100 for 1 h at room temperature coverslips were incubated with primary antibodies diluted in blocking solution for 1 h at room temperature or at 4°C overnight. Alexa Fluor-conjugated anti-mouse or anti-rabbit immunoglobulins (Molecular Probes, Invitrogen) were used as secondary antibodies (1:1000 in PBS/0.1% Triton X-100) and cell nuclei were visualized with DAPI. Fluorescent images were taken using BX61 microscope (Olympus) and processed using CellF software (Olympus).

### Human tissue samples

Human spinal cord paraffin sections from clinically and histopathologically characterized disease and control cases were obtained from the MRC London Neurodegenerative Diseases Brain Bank (Institute of Psychiatry, King’s College, London). Consent was obtained from all subjects for autopsy, histopathological assessment and research in accordance with local and national Ethics Committee approved donation.

### Immunohistochemistry

Mouse tissues were fixed, embedded in paraffin wax and 8 µm thick sections mounted on poly-l-lysine coated slides as described previously (53). Human spinal cord samples embedded in paraffin were cut 7 µm thick. Immunostaining was performed using Elite plus kits (Vector laboratories) and 3,3′-diaminobenzidine (DAB, Sigma) as a substrate. Microwave antigen retrieval in sodium citrate buffer was performed prior to all stainings. For double immunofluorescence, secondary Alexa Fluor-conjugated antibodies (1:1000, Molecular Probes, Invitrogen) were used and nuclei were stained with DAPI. The same microscope, camera and software were used as described above for epifluorescence and fluorescence imaging of stained tissue.

### Primary antibodies

Commercially available primary antibodies against the following antigens were used: FUS (rabbit polyclonal, #ab84078, Abcam; mouse monoclonal, #sc-47711, Santa Cruz; mouse monoclonal, #sc-135911, Santa Cruz; mouse monoclonal, #611385, BD Biosciences); p54nrb/NONO (rabbit polyclonal C-terminal, Sigma); PSP1 (rabbit polyclonal N-terminal, Sigma); PSF (SFPQ, AB2, Sigma); Flag peptide (M2, Sigma); SMN (mouse monoclonal, #610646, BD Biosciences); p80 coilin (mouse monoclonal, #612074, BD Biosciences); Smith antigen (Y12, rabbit polyclonal, #ab3138, Abcam); TDP-43 (rabbit polyclonal, #SAB3500236, Sigma); PML (chicken, a kind gift from Professor Ronald Hay, Dundee); GFP (Living Colours^®^ rabbit polyclonal, #632593, Clontech); p68 (rabbit monoclonal, clone D15E10, Cell Signaling); beta-actin (mouse monoclonal, AC15, Sigma). Primary antibodies were used at 1:1000 dilution for all applications. For staining of human samples antibodies against p54nrb/NONO were used in 1:500 dilution.

### Immunoprecipitation

At 24 h post-transfection, cells were washed in PBS and lysed in ice cold IP buffer (PBS/1% Triton X-100) and left on ice with periodic vortexing for 20 min. Unbroken cells and cell debris were pelleted at 13 000 rpm for 20 min in a сold centrifuge and supernatant collected for IP. Input sample was taken at this point. Cell lysates were preincubated with anti-GFP antibody (Protein Synthesis, clone 3A9) for 30 min followed by incubation with ProteinA/G sepharose beads (GE Healthcare) or with GFP-Trap^®^ agarose beads (ChromoTek) omitting the antibody step, for 2 h at 4°C. Beads were washed twice in ice cold IP buffer, and bound immunocomplexes were eluted from beads by boiling for 10 min at 100°C in SDS–PAGE loading buffer. In the case of ProteinA/G sepharose beads, control samples were prepared by omitting the antibody step. To remove beads, samples were centrifuged at 2000*g* for 2 min. Samples were then analysed by western blotting. For input 10% of final IP sample was loaded.

#### RT-PCR and qPCR

Total RNA was isolated using RNeasy mini kit (Quiagen) and possible DNA contamination removed using RNase free DNase kit (Qiagen). First-strand cDNA synthesis was carried out on 500 μg RNA using SuperScript III reverse transcriptase (Invitrogen) and random hexamers (Promega) according to manufacturer's instructions. Quantitative real-time PCR was run in triplicate on an ABI StepOne™ real-time PCR instrument and data were analysed using StepOne™ Software v2.0 (Applied Biosystems) according to (54). cDNA amount for each gene was normalized to that of GAPDH. Primer sequences used were as follows: FUS—forward: 5′-tctttgtgcaaggcctgggt-3′; reverse: 5′-taatcatgggctgtcccgtt-3′; NEAT1—forward: 5′-cttcctccctttaacttatccattcac-3′; reverse: 5′-ctcttcctccaccattaccaacaatac-3′; GAPDH—forward: 5′-tcgccagccgagcca-3′; reverse: 5′-gagttaaaagcagccctggtg-3′.

### Western blotting

For SDS–PAGE loading buffer was used to lyse cells on dishes, followed by denaturation at 100°C for 5 min. After SDS–PAGE, proteins were transferred to PVDF membrane by semi-dry blotting followed by blocking, incubation with primary and horse radish peroxidase-conjugated secondary (GE Healthcare) antibodies and ECL detection as described previously (55,56). Equal loading was confirmed by re-probing membranes with antibodies against beta-actin.

### Statistics

Statistical analysis was performed with Mann–Whitney *U-*test using IBM SPSS Statistics software (IBM).

## SUPPLEMENTARY MATERIAL

Supplementary Material is available at *HMG* online.

## FUNDING

This work was supported by research grants from the Welcome Trust (075615/Z/04/z) and Russian Federation Program (agreement no. 8829) to V.L.B. H.K.R. was supported by the Cardiff NMHRI 4-year PhD Studentship Programme and T.A.S. by Russian Foundation for Basic Research (grant no. 12-04-31791).

## Supplementary Material

Supplementary Data
