# Supplementary material for: Compromised paraspeckle formation as a pathogenic factor in FUSopathies
Source: Hum Mol Genet. 2013 Dec 11;23(9):2298–312. doi: 10.1093/hmg/ddt622 (PMC3976330; doi:10.1093/hmg/ddt622)
Supplement: Supplementary Data [file supp_23_9_2298__index.html]

Compromised paraspeckle formation as a pathogenic factor in FUSopathies — Compromised paraspeckle formation as a pathogenic factor in FUSopathies — Supplementary Data 

# Compromised paraspeckle formation as a pathogenic factor in FUSopathies

## Supplementary Data

Supplementary Data

**Files in this Data Supplement:**

- Supplementary Data - Docx file
