# Supplementary material for: Compromised paraspeckle formation as a pathogenic factor in FUSopathies
Source: Hum Mol Genet. 2013 Dec 11;23(9):2298–312. doi: 10.1093/hmg/ddt622 (PMC3976330; doi:10.1093/hmg/ddt622)
Supplement: Supplementary Data [file supp_ddt622_ddt622supp.docx]

**
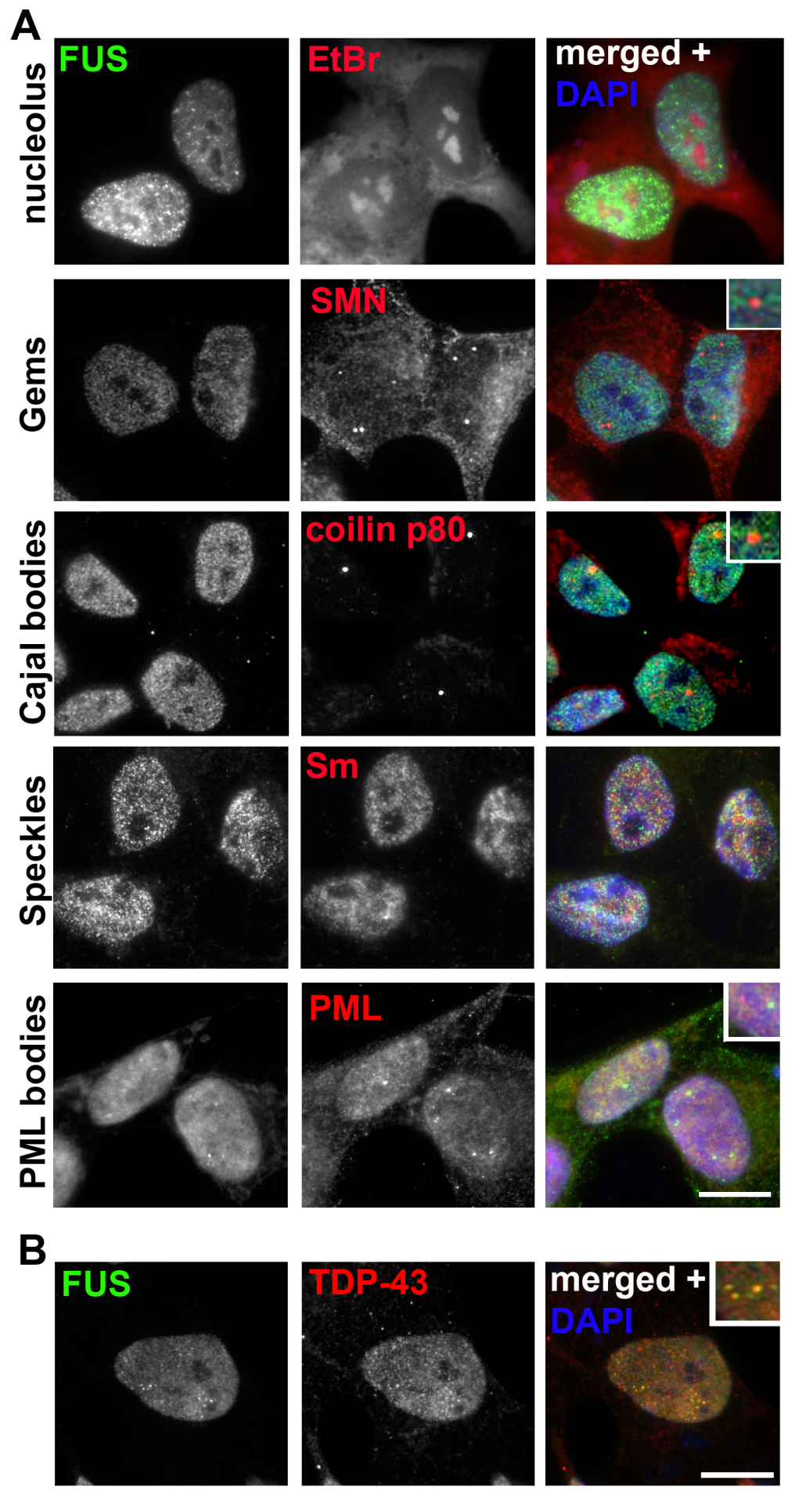
**

**Figure S1. FUS protein and known nuclear bodies in neuroblastoma SH-SY5Y cells. (A)** FUS is excluded from nucleolar regions (ethidium bromide, EtBr), does not localise to Gems (SMN), Cajal bodies (coilin p80) or PML bodies. The protein is moderately enriched in nuclear speckles (Sm antigen, Y12 antibody). **(B)** Endogenous TDP-43 localises to FUS-positive paraspeckles. Scale bar, 10 µm.

**
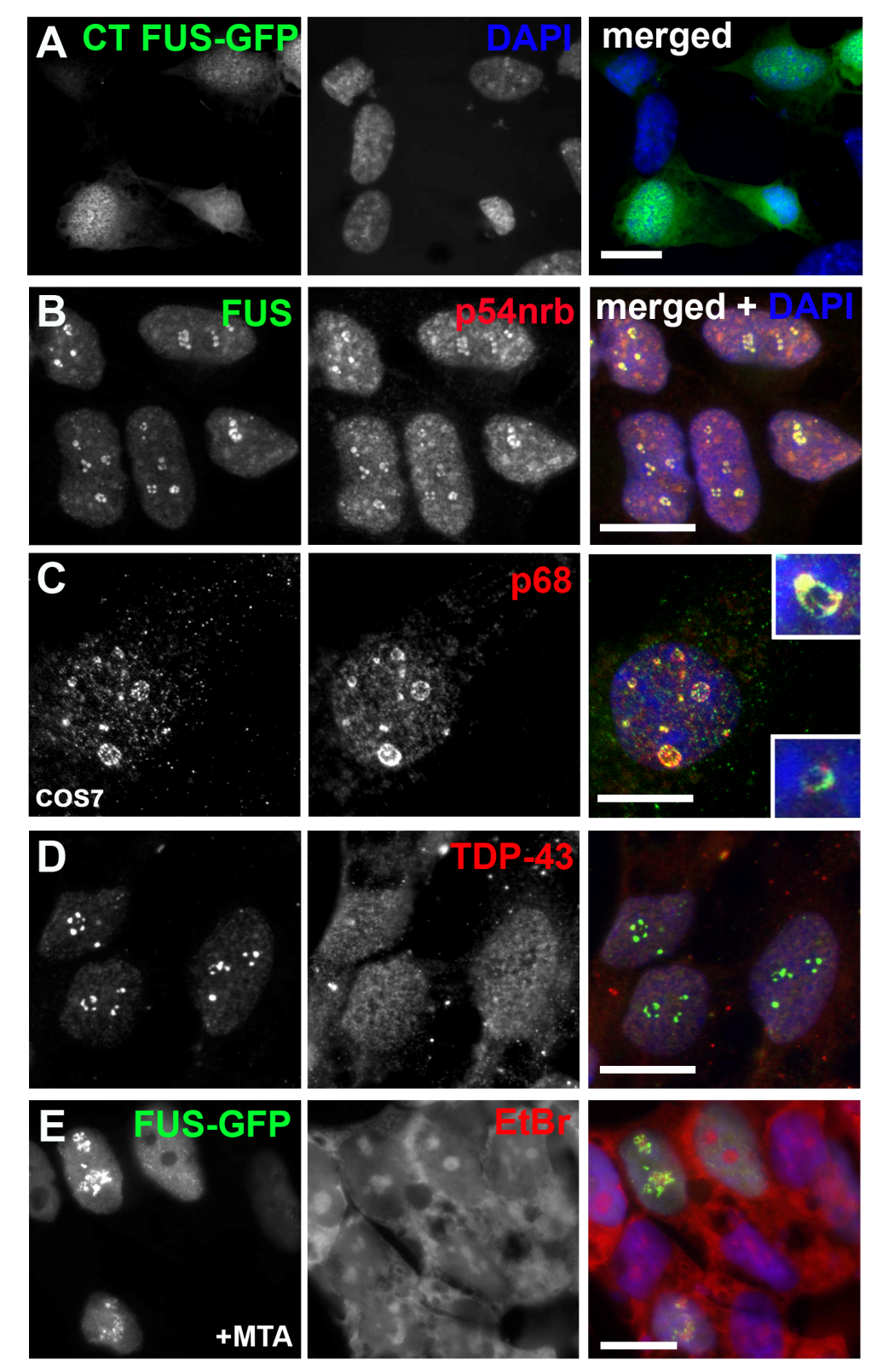
**

**Figure S2. Changes in nuclear distribution of paraspeckle proteins in response to treatment with inhibitors. (A)** GFP-tagged C-terminal part of FUS (FUS CT) is only weakly recruited to nucleolar caps in high expressing cells. **(B)** Nucleolar caps formed by FUS and p54nrb completely coincide**. (C)** FUS and RNA helicase p68 co-assemble complex three-dimensional structures in perinucleolar area consisting of completely overlapping and of completely distinct regions. **(D)** TDP-43 is not targeted to nucleolar caps under conditions of transcriptional arrest. In panels A-D SH-SY5Y (A, B, D) or COS7 (C) cells were treated with actinomycin D for 1.5 hours to inhibit transcription and induce formation of nucleolar caps. **(E)** GFP-tagged FUS is targeted to the perinucleolar region in SH-SY5Y cells subjected to global methyltransferase inhibitor MTA for 24 hours. Scale bars, 10 µm.


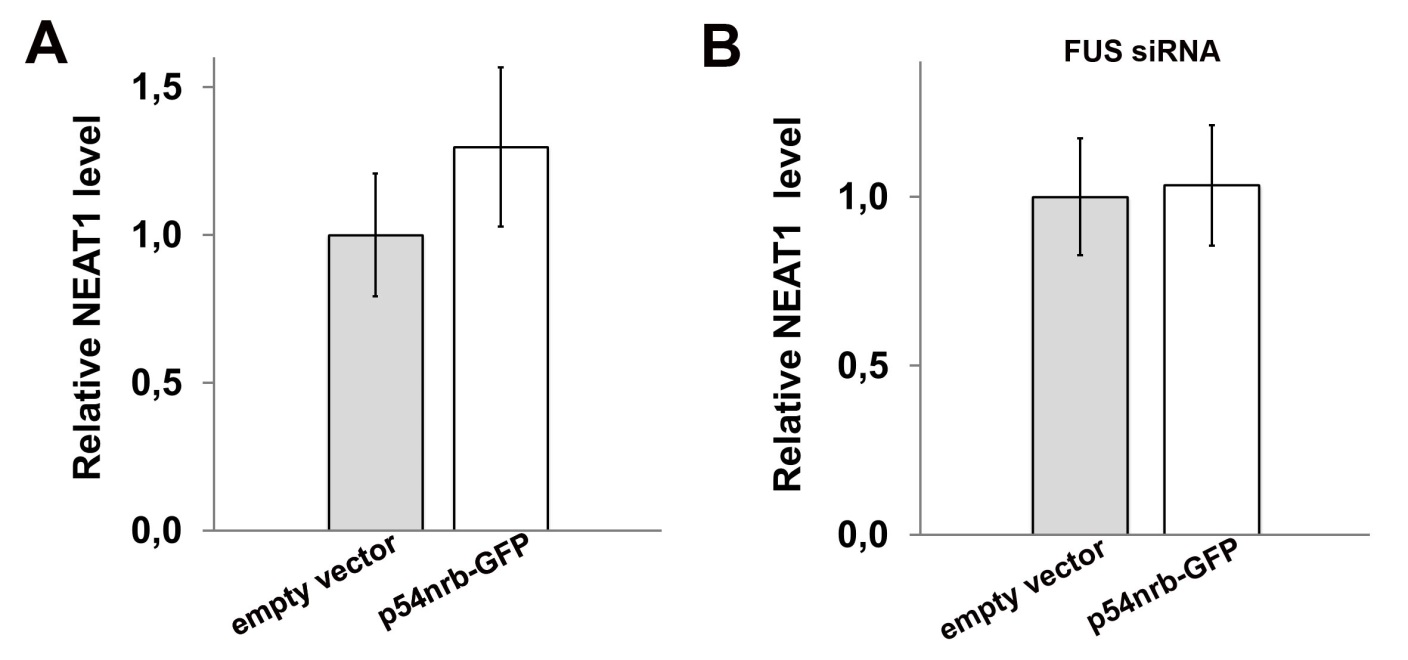


**Figure S3. p54nrb overexpression does not alter NEAT1 levels in naïve cells (A) or cells depleted of FUS protein by siRNA treatment (B).** In A, MCF7 cells were transfected with empty vector to express GFP only or with p54nrb-GFP construct; NEAT1 levels were measured 24 hours post-transfection. In B, MCF7 cells were transfected with FUS siRNA in combination with either empty vector or p54nrb-GFP construct followed by analysis of NEAT1 expression 72 hours post-transfection.

**
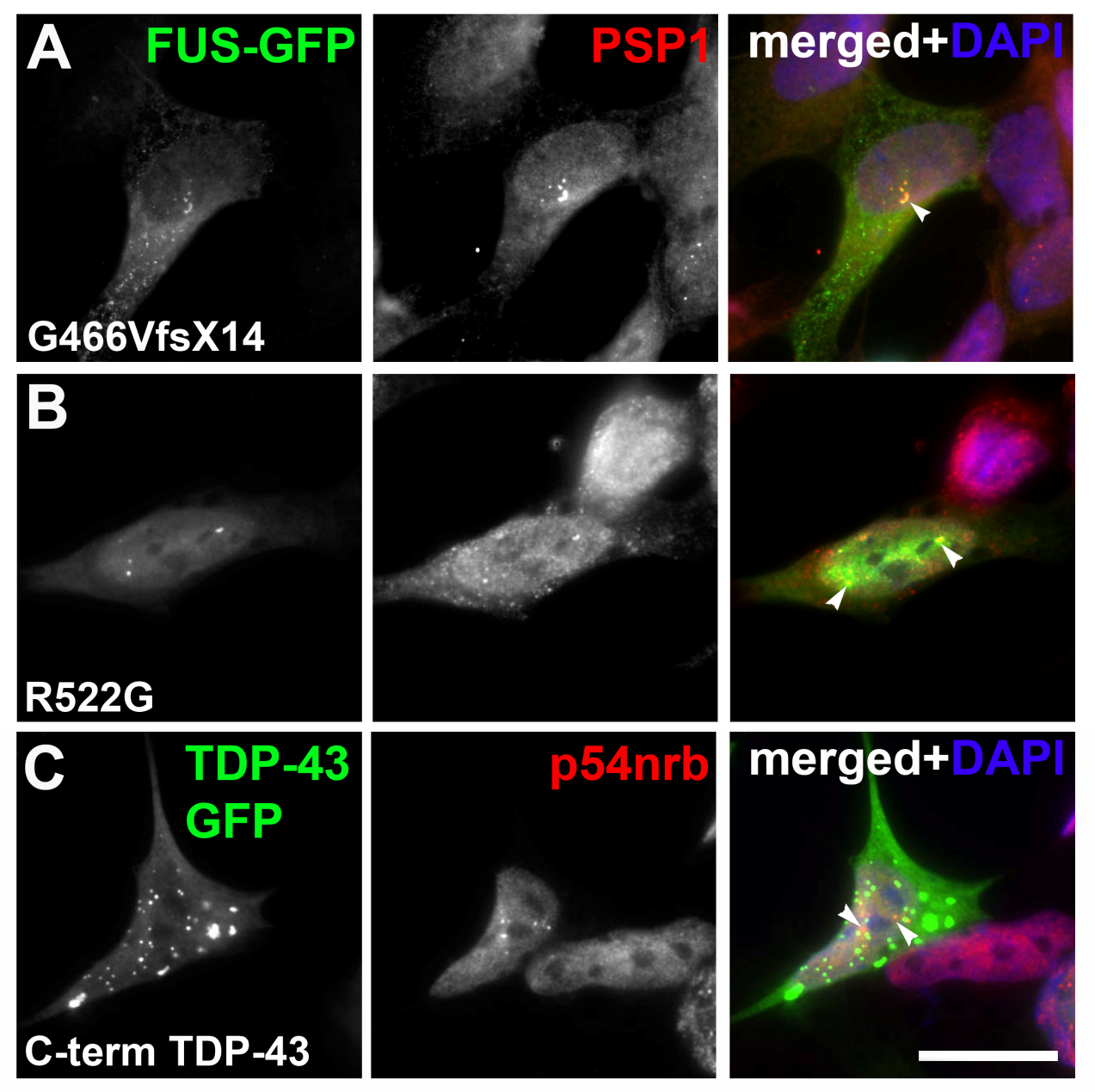
**

**Figure S4. ALS-associated FUS mutants with predominant cytosolic localisation but not 25 kDa C-terminal fragment of TDP-43 are targeted to paraspeckles. (A, B)** ALS-associated FUS variants with disrupted nuclear localisation signal due to C-terminal truncation after amino acid 466 (G466VfsX14) **(A)** or point mutation R522G **(B)** are enriched in paraspeckles in neuroblastoma SH-SY5Y cells. **(C)** Cytoplasmic aggregates formed by GFP-tagged TDP-43 C-terminal fragment of 25 kDa do not recruit p54nrb, and its nuclear aggregates do not colocalise with paraspeckles. Scale bar, 15 µm.


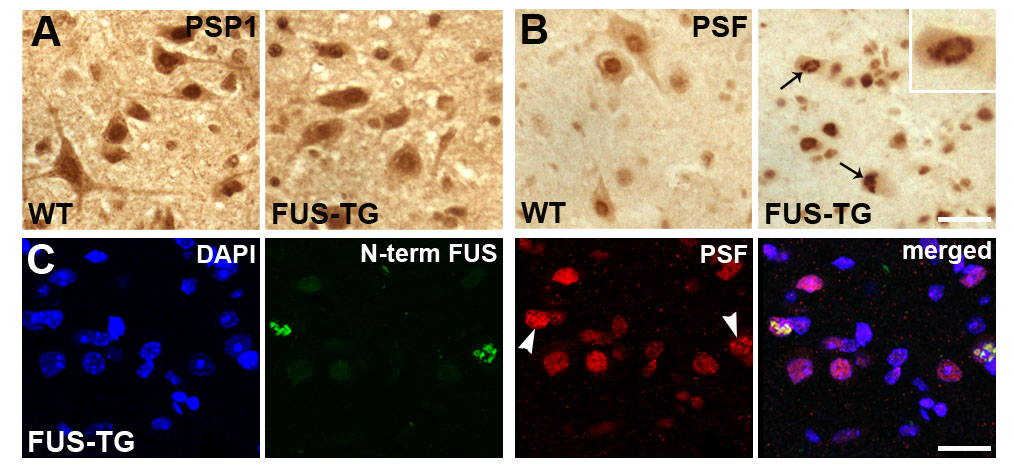


**Figure S5. PSF but not PSP1 is sequestered into nuclear inclusions formed by truncated FUS in spinal neurons of FUS-TG mice. (A)** Immunohistochemical staining of spinal cord sections of wild-type and FUS-TG mice with anti-PSP1 antibody reveals that the protein is present both in the nucleus and in the cytoplasm of large neurons. However, no PSP1-positive inclusions were observed. **(B, C)** PSF protein is predominantly nuclear in both wild type and FUS-TG mice and in neurons of latter animals it is frequently present in nuclear FUS inclusions (arrows in B and arrowheads in C). Both truncated and endogenous FUS were visualised by an antibody recognising an N-terminal FUS epitope (N-term FUS) present in both proteins. Scale bars, A, B - 30 µm; C - 20 µm.
